# Xanthogranulomatous prostatitis: A rare mimicker of prostate adenocarcinoma

**DOI:** 10.1002/ccr3.2610

**Published:** 2019-12-13

**Authors:** Alain Mwamba Mukendi, Sean Doherty, Reena Mohanlal

**Affiliations:** ^1^ Department of Urology Chris Hani Baragwanath Academic Hospital University of the Witwatersrand Johannesburg South Africa; ^2^ Department of Anatomopathology Chris Hani Baragwanath Academic Hospital University of the Witwatersrand Johannesburg South Africa

**Keywords:** diagnostic dilemma, prostate adenocarcinoma, prostatitis, xanthogranulomatous

## Abstract

Xanthogranulomatous prostatitis as mimicker of prostate adenocarcinoma can cause a diagnostic dilemma, as presented in this case. Therefore, alongside histopathology analysis, multiparametric magnetic resonance imaging (mpMRI) would be useful in this situation by identifying and characterizing suspicious prostatic lesions before biopsy thereby supporting current recommendations on the use of mpMRI.

## Graphical abstract

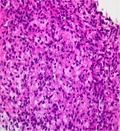

## 1 INTRODUCTION

Xanthogranulomatous prostatitis is an uncommon mimicker of prostate adenocarcinoma clinically, biochemically, and histopathologically. It is a very infrequent histopathological entity. The most common xanthogranulomatous inflammations are seen in the kidneys and gallbladder.^1^ Interestingly, the prostatic one though rare can mimic prostate adenocarcinoma.^2^ We present an unusual case of diagnostic dilemma in which xanthogranulomatous prostatitis is diagnosed following an initial diagnosis of adenocarcinoma of prostate was made a few years ago.

## 2 CASE REPORT

**Figure 1:**
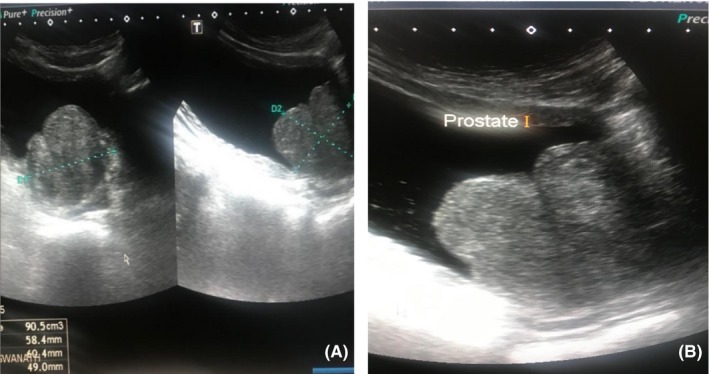
A and B, Transabdominal ultrasound depicting an heterogenous lobular prostate gland measuring 90 cm^3^

**Figure 2:**
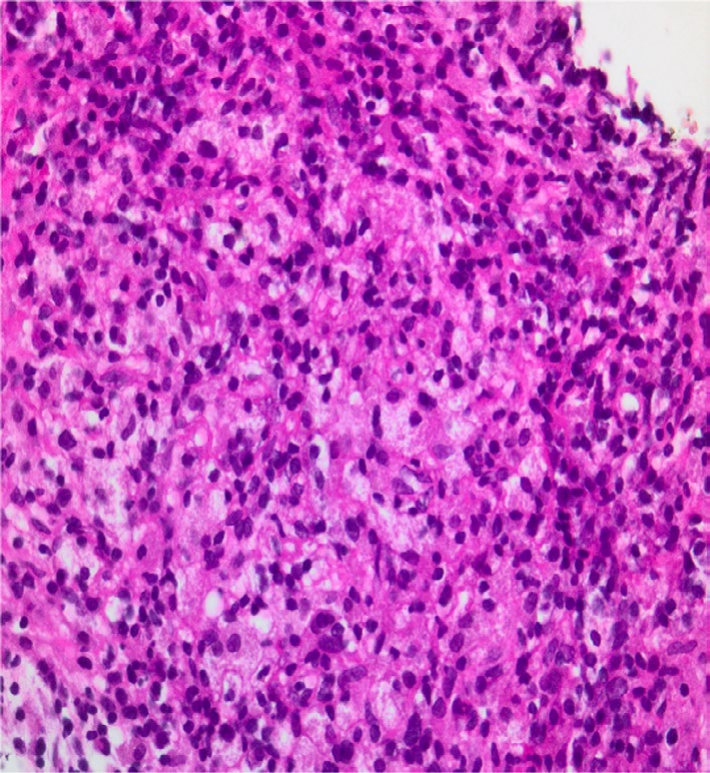
Histological examination showing numerous lymphocytes and epithelioid and foamy histiocytes in keeping with a granulomatous prostatitis (original magnification × 400)

A 71‐year‐old male diagnosed in 2010 with prostate adenocarcinoma, gleason score of 3 + 3 = 6, PSA of 4 µg/L and no metastases on bone scan. Patient declined radical prostatectomy and opted for active surveillance. He was also known with chronic kidney disease secondary to hypertension and diabetes and had no previous or current history of tuberculosis. He has been voiding well on doxazocin XL 8 mg po nocte. Due to progressive rising in PSA from 4 (2010) to 9.5 µg/L(2018), a repeat prostate biopsy was planned. Digital rectal examination revealed an enlarged firm and nodular prostate (T2c). Transabdominal ultrasound outlined a lobular heterogeneous prostate measuring 90 cm^3^ (Figure 1A,B). Uroflowmetry did not show any features of bladder outlet obstruction with a maximal flow of 15.1 mLs/s, and there was no post void residual volume on ultrasound. In the absence of multiparametric magnetic resonance imaging (mpMRI) facility, systematic transrectal ultrasound guided sextant biopsy was performed and with tissue fragmentation a total of 20 cores were sent. The histopathology diagnosis was of an extensive xanthogranulomatous prostatitis (Figure 2) with all the cores involved. The Ziehl Neelsen on the specimen was negative for acid‐fast bacilli.

## 3 DISCUSSION/CONCLUSION

Xanthogranulomatous prostatitis is indeed a rare entity and rare mimicker of prostate adenocarcinoma.^1^, ^2^ The clinical and sonographic findings in this case were highly suggestive of prostate adenocarcinoma as reported on the initial histopathology assessment. Unexpectedly, xanthogranulomatous prostatitis turned out to be the diagnostic conclusion on the repeated biopsy specimens 9 years down the line.

In this prostatic entity, a large amounts of histiocytes (foamy macrophages) are observed accompanied by multiple plasma cells and lymphocytes. The presence of histiocytes may cause confusion and may be diagnosed as hypernephroid pattern type adenocarcinoma of the prostate.^2^ Although it is still very challenging to distinguish granulomatous prostatitis from adenocarcinoma even on multiparametric MRI (mpMRI), the evidence of diffuse changes involving more than 50% of the prostatic gland associated with infiltration of periprostatic fat or extracapsular extension, the presence of large areas of nonenhancement corresponding with caseous abscess and areas of rim enhancement may suggest the diagnosis of xanthogranulomatous prostatitis.^3^, ^4^

The presence of extensive xanthogranulomatous prostatitis on the repeated biopsy specimens may indicate diffuse changes within the prostate which may involve a significant volume of the gland. Unfortunately, mpMRI facility is not yet available in our institution. The mpMRI often spots areas under‐sampled by the systematic transrectal ultrasound guided biopsy and is very helpful in characterizing lesions.

Histopathology analysis including immunohistochemistry staining will definitely be helpful in distinguishing xanthoma cells from carcinoma when facing histopathological dilemma as xanthoma cells can appear like high‐grade prostatic carcinoma.^5^ Immunohistochemistry staining in xanthogranulomatous prostatitis would be negative for broad spectrum cytokeratins and positive for CD68.^6^, ^7^ In this case, CD68 and cytokeratins were not done simply because the pathologist was convinced of the presence of foamy macrophages.

In the literature, it may be described alone or with another concomitant condition. Xanthogranulomatous prostatitis has been reported associated with prostatic abscess.^1^ Ultrasound in this case did not reveal any abscess and the patient did not have any signs or symptoms suggestive of an abscess. Another report in the literature presented a case of prostato‐rectal fistula from xanthogranulomatous prostatitis and stated that this complication may occur either spontaneously by prostatic abscess perforating into rectum, or after a transurethral resection of prostate (TURP) in patients with the condition.^7^

Management of xanthogranulomatous prostatitis is mainly conservative (alpha blockers, corticosteroids). Surgical management (TURP or open prostatectomy) is indicated if symptoms are significant and/or failed conservative management.^7^ Our patient did not require any surgical management as his symptoms were well controlled on medical therapy.

In conclusion, xanthogranulomatous prostatitis as mimicker of prostate adenocarcinoma can cause diagnostic dilemma as presented in this case. However, alongside histopathology analysis, multiparametric magnetic resonance imaging (mpMRI) would be useful in this situation by identifying and characterizing suspicious prostatic lesions prior to biopsy hence supporting current growing recommendations on the use of mpMRI as diagnostic tool.^8^

In this particular case scenario, the dilemma may have been resolved if we had mpMRI available in our institution to characterize the lesions and probably aid in diagnosis of either combined pathologies (xanthogranulomatous prostatitis and prostate adenocarcinoma) or only xanthogranulomatous prostatitis with the possibility of the previous histopathology assessment being incorrect.

## CONFLICT OF INTEREST

None declared.

## AUTHOR’S CONTRIBUTIONS

AMM: involved in substantial contributions to conception and design of the case report; involved in acquisition of data; drafted the manuscript; involved in critical revision for important intellectual content; and approved the final version. SD: involved in critical revision for important intellectual content and approved the final version. RM: involved in substantial contributions to acquisition of data and drafting of part of the manuscript.

## CONSENT

Written informed consent was obtained from the patient for publication of this manuscript and accompanying pictures. A copie of the written consent is available for review by the Editor‐in‐Chief of this journal.
